# Surf and turf: predation by egg-eating snakes has led to the evolution of parental care in a terrestrial lizard

**DOI:** 10.1038/srep22207

**Published:** 2016-02-26

**Authors:** David A. Pike, Rulon W. Clark, Andrea Manica, Hui-Yun Tseng, Jung-Ya Hsu, Wen-San Huang

**Affiliations:** 1 School of Marine and Tropical Biology, James Cook University, Australia; 2 Department of Biology, San Diego State University, USA; 3 Department of Zoology, University of Cambridge, CB2 3EJ UK; 4 Department of Biology, National Museum of Natural Science, Taiwan; 5 Department of Life Sciences, National Chung Hsing University, Taiwan

## Abstract

Animals display a great diversity of parental care tactics that ultimately enhance offspring survival, but how such behaviors evolve remains unknown for most systems. Here, we studied the evolution of maternal care, in the form of nest guarding, in a single population of long-tailed sun skink (*Eutropis longicaudata*) living on Orchid Island (Taiwan). This species typically does not provide protection to its offspring. Using a common garden experiment, we show that maternal care is genetically determined in this population. Through field manipulations, we demonstrate that care provides a significant increase in egg survival on Orchid Island by reducing predation from egg-eating snakes (*Oligodon formosanus*); this predator is not abundant in other populations of the lizard, which do not display parental care. Finally, using extensive field surveys, we show that the seasonal availability of green sea turtle (*Chelonia mydas*) nests is the cause for the high abundance of snake predators on Orchid Island, with the snakes consuming lizard eggs when green turtle eggs are not available. Together, these lines of evidence provide the first full demonstration of how predation can trigger the evolution of parental care in a species derived from a non-caring ancestor.

Parental care, defined as any form of behavior that increases the fitness of offspring by providing protection or food, is an important feature of most endotherms (birds and mammals) and a number of ectotherms (ex. reptiles, amphibians, fishes, and insects). Across many taxa, a wide diversity of parental care strategies have evolved, ranging from relatively primitive nest-guarding behaviors to more complex interdependency between parents and offspring, such as active food provisioning to offspring^1^,^2^,^3^,^4^,^5^. Even though parental care is known to have arisen many times independently in disparate animal lineages^3^,^6^,^7^,^8^,^9^,^10^,^11^, studying its evolution from a non-caring ancestor empirically is challenging, since the expression of care tends to be conserved within each species. For this reason, most work on this topic has relied on theoretical modelling (ex.^12^,^13^,^14^,^15^,^16^,^17^), or on investigating the predictors of how much care parents provide, rather than on the consequences of providing no care at all.

For many taxa, the driving force behind the evolution of parental care is thought to be the protection of young or eggs from predation^18^. Predators have been shown to drive the evolution of many traits, including avoidance (e.g., reduced feeding and activity, and refuge use) (ex.^19^,^20^), crypsis^21^, aposematism^22^,^23^, mimicry^24^, and masquerading (visual resemblances to inedible objects)^25^. Although predation pressure (i.e., high offspring mortality in the absence of care) is frequently cited as the most likely factor driving the evolution of caring behavior from a state of no care^1^,^3^, there is little direct evidence of this relationship in natural systems (see also Brown *et al.* 2010^26^). This is because unraveling the evolutionary origins of care requires comparing populations that differ in the expression of parental care—for example, by comparing the ecological attributes and selective pressures acting on a single species that shows parental care in some populations, but not others. Although some species show geographic variation in the type and intensity of parental care provided, virtually all species that show parental care always express the behavior in some form^1^,^27^.

One species that does exhibit intraspecific variation in parental care is the long-tailed sun skink (*Eutropis longicaudata*). Mothers from a single population on Orchid Island, off the coast of Taiwan, attend their egg clutches after laying, aggressively warding off egg predators, whereas lizards from other populations always abandon egg clutches immediately after laying^28^. Predation by the egg-eating snake *Oligodon formosanus* is the most likely cause for the evolution of care on Orchid Island, as the abundance of this predator is unusually high at that location^28^. The reason for such high snake abundance is unknown, even though anecdotal evidence suggests that nearby green sea turtle eggs (*Chelonia mydas*) might be an important food source for this snake^29^. Thus, the long-tailed sun skink provides the opportunity to empirically investigate the evolution of parental care from a non-caring ancestor.

Here, we show that parental care on Orchid Island is genetically determined, and quantify the role of predation in favouring this behaviour on Orchid Island, as well as investigating why predation pressure is unusually high at this location. We do so by first performing a common garden experiment to study the expression of anti-snake behavior in captive lizards from Orchid Island as well as from two non-caring populations (Green Island and mainland Taiwan) which show low levels of genetic differentiation from one another^28^. We then investigate the sources of egg mortality in unattended nests in these three populations, comparing nests on Orchid Island from which guarding mothers were removed to nests at the other two locations and nests that were artificially protected from snake predators all three locations. Finally, we investigated the annual and seasonal relationship between green sea turtle (*Chelonia mydas*) nesting, egg-eating snake population dynamics and density, and predation rates on long-tailed sun skink nests on Orchid Island, to understand the role of this trophic link between terrestrial and marine habitats in the evolution of lizard maternal care.

## Results

### Genetic basis of parental care on Orchid Island

Our common garden maternal attendance experiment used 10 captive-raised females with eggs from Orchid Island, 14 from Green Island, and 12 from the mainland Taiwan site, none of which had prior experience nesting or encountering snakes. When confronted with egg-eating snakes, all 10 females from Orchid island violently attacked snakes, thereby demonstrating egg-guarding behaviour, but none of the females from the other two sites did so, indicating that the guarding behaviour has a genetic basis despite the low level of overall genetic differentiation among these populations^28^.

### Hatching success of lizard eggs and sources of predation

**Figure 1:**
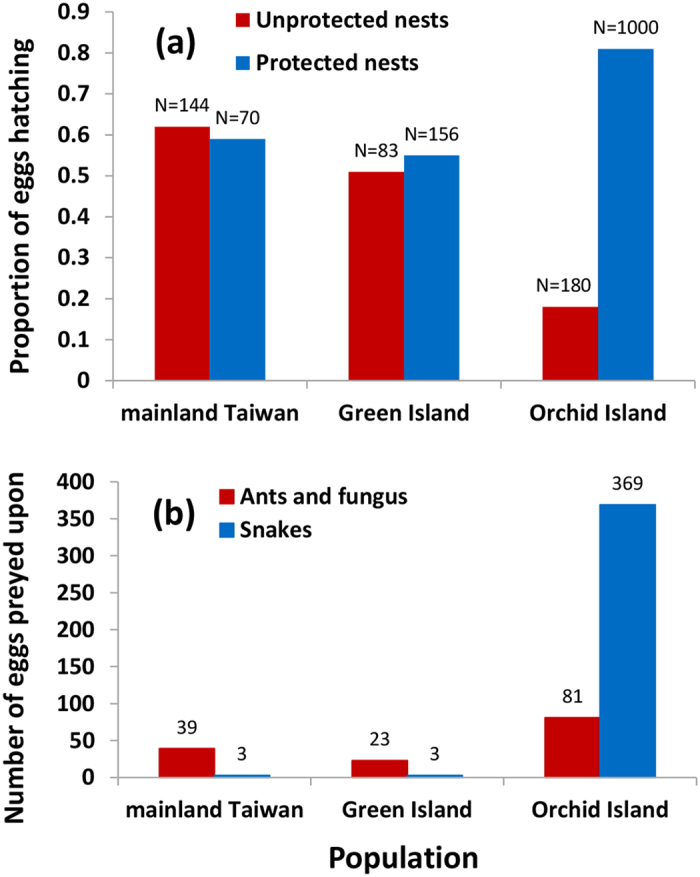
Predation rates of lizard nests and associated predators. Proportion of long-tailed sun skink eggs hatching from three populations without any form of nest protection ((**a**) for the mainland, Green Island, and Orchid Island, n = 108, 60, and 450 eggs) and when nests were protected by predator-excluding mesh (n = 1000, 70, and 156 eggs). Known causes of egg mortality, expressed as the numbers of eggs preyed on by snakes or ants and fungi (**b**).

Hatching success of lizard eggs in the absence of maternal care was lowest on Orchid Island (18%), and much higher in the two populations lacking maternal care (62% at Green Island, and 52% at the mainland Taiwan site; χ^2^_2_ = 106.74, *P *< 0.0001; Fig. 1a). There was no significant difference among the sites in key environmental parameters, such as nest temperatures (mean ± SE: Orchid Island = 30.0 ± 0.30 °C [n = 125]; Green Island = 30.3 ± 0.30 °C [n = 45]; mainland = 30.8 ± 0.21 °C [n = 248]; *F*_2,415_ = 1.57, *P* = 0.21) and relative humidity (Orchid Island: 91.0 ± 1.9% [n = 125], Green Island: 90.9 ± 2.6% [n = 45]; mainland Taiwan: 91.5 ± 2.4% [n = 248]; *F*_2,415_ = 0.89, *P* = 0.76), which therefore do not explain differences in hatching success among populations.

The causes of lizard egg mortality differed significantly among populations (χ^2^_2_ = 143.07, *P *< 0.0001), with snakes preying upon most eggs on Orchid Island, but ants and fungal infections causing most mortality at the other two sites (Fig. 1b). Even when scoring data from eggs which died from unknown causes as snake predation, Orchid Island had the highest snake predation rates (comparing all three sites: χ^2^_2_ = 89.60, *P *< 0.0001; comparing only the two sites with highest putative snake predation rates: Orchid Island vs. mainland Taiwan, χ^2^_1_ = 86.30, *P *< 0.0001).

Experimentally excluding vertebrate predators from accessing lizard eggs substantially increased hatching success rates on Orchid Island (n = 1000 eggs, χ^2^_1_ = 200.89, *P *< 0.0001; Fig. 1a), but had no significant effect at the other two sites (mainland Taiwan: n = 70 eggs, χ^2^_1_ = 0.31, *P* = 0.58; Green Island: n = 156 eggs, χ^2^_1_ = 0.17, *P* = 0.68; Fig. 1a). This confirms that Orchid Island is the only population for which maternal nest-guarding would provide a fitness benefit (Fig. 1a). Consequently, high predation pressure from egg-eating snakes likely led to the initial development of maternal nest-guarding on Orchid Island.

### Snake density close to lizard nests

**Figure 2:**
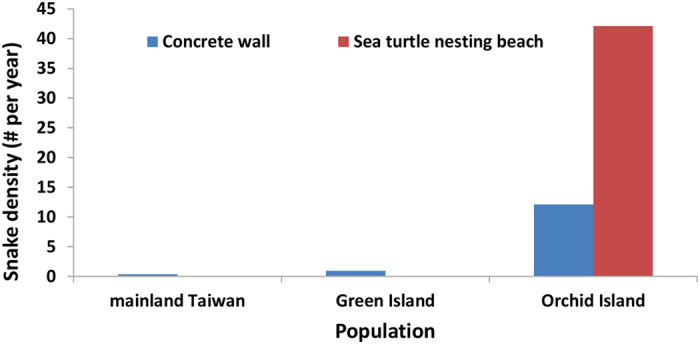
Snakes observed on concrete walls on Green island and Orchid Island when turtle eggs on Orchid Island are available (May–October) and not available (November–April) from 1997–2008.

Given the predominant role of snake predation at Orchid Island, we surveyed the abundance of this predator on concrete walls, where lizards nests, at our three locations between 1997 and 2008. The snake population density (number of snakes per year) was over ten times higher at Orchid Island (Mean ± SE = 12.1 ± 0.95) relative to the other two sites (Mean ± SE = 0.91 ± 0.71 and 0.36 ± 0.45 in Green Island, and mainland Taiwan, respectively, χ^2^_2_ = 6.56, *P *= 0.0001; Fig. 2).

### The role of turtle nesting in driving lizard abundance

**Figure 3:**
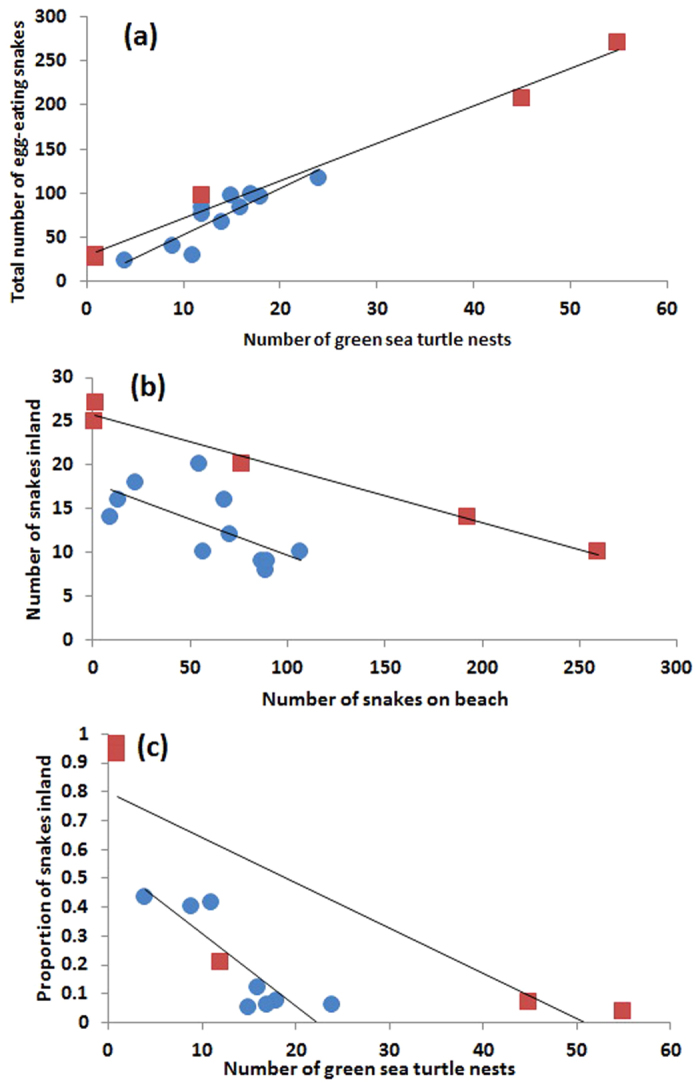
Relationships between sea turtle nesting and egg-eating snake abundance on Orchid Island, Taiwan. Egg-eating snake abundance depends upon sea turtle nest availability (**a**). When more egg-eating snakes are at the sea turtle nesting beach, fewer are found inland (**b**). Ultimately, there is a negative relationship between sea turtle nest availability and the proportion of snakes inland (**c**). Circles show annual values (n = 11) and squares show monthly values (n = 5).

Orchid Island is a major nesting site for green turtles in Taiwan, and provides a potential seasonal source of food for egg-eating snakes. To determine whether this food source is an important driver of the snake density, and thus of the predation pressure experienced by lizards, we surveyed the abundance of egg-sneaking snakes at Orchid Island, running monthly surveys and distinguishing between snakes captured in inland habitat where lizards nest, and beach habitat where sea turtles nest. Over the whole island (pooling estimates from inland and beach sites), we found snake density to be highest in years of greater sea turtle nesting effort (Fig. 3a; *R*^2^ = 0.79, *F*_1,9_ = 33.54, *P *< 0.0001), and within each year density peaked in the months (May-October) when sea turtles nest (*R*^2^ = 0.99, *F*_1,3_ = 224.42, *P *< 0.0001). By contrast, there was no relationship between annual sea turtle and lizard nest abundances (n = 8 years, Pearson’s correlation r = 0.06, *P* = 0.88), nor between snake and lizard nest abundance (r = 0.11, *P* = 0.64); thus the relationship between sea turtle nesting and snake abundance is driven by processes that are different from those driving lizard nesting.

Snakes were more abundant on the beach than inland, and the strong negative relationship between snake abundances in the two habitats suggests a dynamic population that readily migrates between the beach and inland sites (annual: *r *= −0.66, *F*_1,12_ = 8.99, *P* = 0.011; monthly: *r* = −0.99, *F*_1,3_ = 176.34, *P *< 0.001; Fig. 3b). Mark-recapture of individual snakes support this conclusion: 72% of recaptured snakes moved between the beach and inland sites (beach: n = 463 individual snakes marked, of which 109 were recaptured, and 78 of those changed sites). Additionally, we found a negative relationship between sea turtle nest availability and the proportion of snakes inland (annual: *r* = −0.83, *F*_1,9_ = 20.77, *P* = 0.001; monthly: *r* = −0.86, *F*_1,3_ = 8.43, *P* = 0.06; Fig. 3c), implying that sea turtle eggs are an important food resource for snakes.

**Figure 4:**
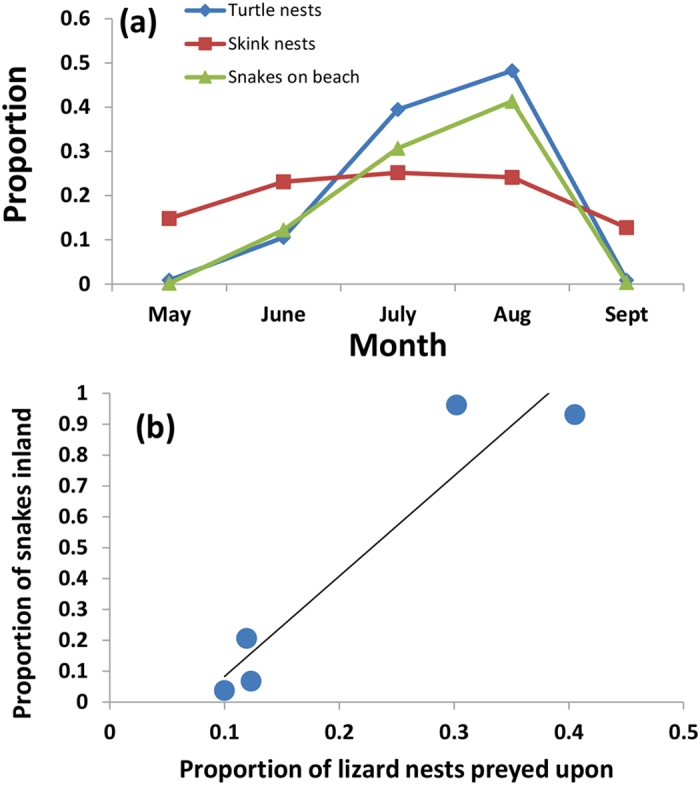
Seasonal patterns of nesting activity and nest predation on Orchid Island, Taiwan. Sea turtle nesting is highly seasonal and coincides with egg-eating snake abundances at the beach, whereas lizard nesting occurred in each month ((**a**) n = 5). Proportions represent the number of monthly observations as a function of the total number. In May and September, >99% of snakes are inland, at the lizard nesting site. Predation rates of lizard nests are highest when proportionately more snakes are inland (**b**).

In contrast to the highly seasonal availability of turtle eggs, lizard nesting was relatively constant in each month, but with slightly fewer nests laid during May and September (Fig. 4a). The migration of snakes to target sea turtle nests resulted in snakes being more abundant inland when fewer lizard nests were available (*r* = −0.81, *F*_1,3_ = 12.68, *P* = 0.038; Fig. 4a). Annual predation rates of lizard nests by snakes remained stable on Orchid Island (n = 390 nests; 10.2–15.6% annual predation from 2000–2008), but monthly predation rates were highest when more snakes were inland (*r* = 0.95, *F*_1,3_ = 28.13, *P* = 0.013; Fig. 4b). The positive relationship between snake abundances inland and lizard-egg predation rates coincides with a decrease in sea turtle nest availability (Fig. 3c). This establishes a clear pattern of foraging driven by snakes moving between sea turtle nests on the beach and lizard nests inland when sea turtle nests are unavailable.

## Discussion

Egg guarding by mother lizards on Orchid Island has a genetic basis, despite the low level of genetic differentiation among lizard populations and lack of maternal care in other populations^28^. This finding underscores the fact that major differences in reproductive strategies can evolve rapidly and with minor genetic changes. The evolution of this trait seems to be in response to the unusual predation pressure experienced at this location, where egg-eating snakes are very abundant and account for the majority of egg mortality. In other lizard populations, however, egg-eating vertebrate predators have a very limited impact on reproductive success^30^. High abundances of egg-eating snakes at Orchid Island are the consequence of green sea turtle nesting; whilst sea turtle eggs seem to be the major source of food for the snakes, their seasonal availability means that snake predation is a major threat to lizard eggs, driving the evolution of care in this population.

We provide comprehensive evidence that the evolution of maternal care (in the form of egg guarding) on Orchid Island is a response to egg predation by snakes. Guarding is highly effective, since lizards are large enough to aggressively attack snakes, but the snakes are obligate egg predators and thus poses no physical threat to the adult lizards^31^ (Supporting information, Video S1). The absence of other predators that can also feed on adult lizards on Orchid Island might have aided the evolution of this behaviour (Supplementary Table S1), but given the results of our manipulations in which snakes were excluded from artificial nests, it seems that the benefit of care at other locations is simply absent (or negligible). Thus, the evolution of care on Orchid island is a combination of a very high benefits (accrued only because of the very high density of egg-eating snakes), and relatively limited costs (at least in terms of survival, variation in body condition, fecundity, or time to laying a subsequent clutch with respect to egg attendance time)^32^.

There is evidence that marine animals, such as sea turtles, can sometimes provide seasonal influxes of nutrients to terrestrial communities that can exert significant influence upon evolutionary dynamics on the structure and dynamics of terrestrial communities, even if they are extrinsic to those communities for most of their life histories^33^,^34^. The annual migration of wild salmon (*Oncorhynchus* spp.) from the North Pacific Ocean into freshwater streams and lakes, where they spawn and die, is possibly the best-studied example of this phenomenon. Adult salmon deliver nutrients to freshwater and terrestrial ecosystems and support the populations of many animal species (e.g. grizzly bear)^35^ and in turn, influence riparian structure and dynamics^33^,^36^. These findings illustrate the complexity of interspecific interactions across ecosystems, and the possible cascading effects on the ecology and life-history of individual species.

We cannot determine when egg guarding may have evolved in skinks nesting on Orchid Island. The concrete barriers used as nesting sites by most lizards on Orchid Island have only been present since 1995. Even though the resulting concentration of nests on this artificial substratum might have made the nests more attractive to egg-eating snakes, it seems unlikely that the trait has become fixed in the population within such a short period of time. It seems more likely that the seasonal nature of turtle nesting has imposed a great evolutionary pressure on the lizards to start defending nests, and this might have been exacerbated since the construction of the concrete barriers. The low level of genetic differentiation between Orchid Island and neighbouring populations makes this species a prime target to look for the genetic mutations that might underlie guarding behavior^28^. Whether there is a “parental gene” remains to be seen, but here we have shown that genetically based parental care can arise and become fixed in a population in response to sufficient predation pressure on the young.

## Methods

### Study system and species

We studied a widely distributed tropical lizard (the long-tailed sun skink, *Eutropis longicaudata*) that may be unique among vertebrates in that only a single population is known to display parental care. This species typically nests in natural grassland/lowland rainforest ecotones where eggs are buried beneath rocks and abandoned immediately after nesting^37^. Thus, parents usually provide no further care for the offspring, as is also the case for most oviparous (egg-laying) lizards^38^. However, female long-tailed sun skinks living on Orchid Island, Taiwan, nest in drainage holes of concrete retaining walls (the warmer temperatures of the concrete wall enhance embryonic development^37^) and remain with their eggs during incubation to actively protect them from predation by egg-eating snakes (*Oligodon formosanus*), which only eat eggs, and not lizards^38^,^39^. Female lizards adjust the duration of egg-guarding according to the risk of egg predation posed by these snakes^32^,^39^. When egg-eating snakes are rare, females leave the nest early in incubation, but when snakes are abundant females continue defending the nest until the eggs hatch^39^ (Supplementary Fig. S1).

The Taiwan kukri snake (*Oligodon formosanus*) is an obligate egg-eater that primarily consumes soft-shelled reptile eggs^31^. On Orchid Island (Taiwan), these snakes primarily consume green sea turtle eggs (*Chelonia mydas*)^37^ and long-tailed sun skink eggs (*Eutropis longicaudata*) (28,37,38; Supplementary Fig. S1). There are substantial advantages to a sea turtle egg diet. Sea turtle eggs provide an unusually abundant and long-lasting food resource for snakes^37^. Individual sea turtle eggs are substantially larger (averaging 43 g each with clutches averaging 105 eggs^40^) than entire lizard clutches (10 g for an average clutch of 6 eggs^37^,^38^) and sea turtle eggs incubate for twice as long as do lizard eggs (averaging 55 days vs 26 days^32^,^40^). Consuming turtle eggs also has important associated costs, due to strong intraspecific competition for this resource. Individual snakes will defend food resources, i.e. turtle nests, by biting the tail of competitors; these attacks are more costly for males than female snakes because the hemipenes are housed in the tail and male reproductive ability could be severely compromised by bites from conspecifics^37^. Although lizard eggs are much smaller (and thus of lower nutritional value), consuming them has much lower costs to snakes (Supplementary Fig. S1).

### Genetic basis of parental care on Orchid Island

During 2011–2014 we collected 100 *Eutropis longicaudata* eggs from populations on each of the Taiwan mainland, Green Island, and Orchid Island, and raised them in captivity to determine if egg-guarding behavior has a strong genetic basis (Supplementary Fig. S2). We built a nesting site environment in our laboratory similar to field nesting sites, with plastic drainage pipes placed in glass terraria (85 × 45 × 30 cm), and maintained temperatures within 26–30 °C^37^. When the eggs hatched and lizards grew to adult size (18 months after hatching), we mated males and females from the same population. We removed the male after observing copulation, and left the pregnant females in their cage. After females laid eggs, we put egg-eating snakes (*Oligodon formosanus*) into the cage, and documented any egg-guarding behavior of the mother lizards collected from the wild. Because anti-snake behaviors were displayed dichotomously (i.e., individuals either exhibited clear violent reactions to the snake, attacking and biting it repeatedly, or ignored the snake^38^), we simply scored lizards as either exhibiting egg-guarding or not.

### Field methods

Our study site on Orchid Island, Taiwan (22°02′N, 121°34′E) is a 110 m long green sea turtle nesting beach with a long-tailed sun skink nesting site located 600 m inland (Supplementary Fig. S2). The sea turtle nesting area is a sandy beach, which allows sea turtles to bury their eggs belowground. The lizard nesting site consists of a 2 km-long concrete retaining wall designed to prevent erosion. Plastic drainage holes (600 holes/km, 10 cm wide × 120 cm long) running through the walls are used by lizards for nesting; lizards lay eggs inside the holes where they remain exposed to predators throughout incubation^32^,^38^.

During our study, sea turtle eggs were available for snakes to consume from 1997–2008. After 2008, most sea turtle nests at Orchid Island were relocated for conservation purposes (due to high levels of snake predation) and thus were no longer available for consumption by snakes during July and August, the peak nesting months.

We surveyed sea turtle nesting beaches six times nightly from May-October 1997–2008 and recorded all sea turtle nests laid and captured each snake observed. Snakes were individually marked with a PIT tag inserted between the trunk muscles and skin. We surveyed the inland lizard nesting site every six hours (4 times daily) from May-October 1997–2008 to monitor snake and lizard numbers, and snake depredation rates of lizard nests^28^. We individually marked snakes using microchips^41^, and used the number of unique individuals captured monthly and annually for analysis. We studied snake: (1) abundances at the sea turtle nesting beaches, (2) abundances at the lizard nesting areas, (3) movements between these two foraging areas, (4) predation of lizard eggs, and (5) snake density (numbers captured per year). In addition to the population on Orchid Island, we studied two other sun skink populations nesting inside retaining walls, and snake densities on the concrete walls where the lizards nest, but which do not have sea turtles nesting nearby: one on mainland Taiwan (22°42′N, 120°38′E) and the other on Green Island (22°39′N, 121°29′E; Supplementary Fig. S1). As with the Orchid Island site, we surveyed these sites at six hour intervals from May-October 1997–2008, and individually marked snakes, recorded lizard nest temperatures/humidity, scored whether lizards guarded nest-sites, and documented the hatching success rates of lizard eggs.

Across all sites, the main causes of lizard egg failure are predation (by snakes or ants^32^,^41^,^42^,^43^) and fungal infection^32^,^38^. We inferred predation by egg-eating snakes when the entire clutch was consumed^32^; ants were directly observed consuming individual eggs^42^,^44^; and fungal infections were visually observed on eggs that failed to hatch^32^. We determined the relative effectiveness of maternal care in each population by removing attending females and then covering a subset of nest holes with plastic mesh (with 3 × 3 mm holes) to experimentally exclude snake predators from accessing lizard eggs, but allowed ants and fungi passed through the nets. We recorded hatching success rates when these predators both could (controls) and could not (vertebrates experimentally excluded) access eggs.

All work was performed in accordance with animal ethics protocols approved by the Taiwanese Wildlife Conservation Act by the Forestry Bureau, Council of Agriculture, Taiwan, and the Taiwanese National Museum of Natural Science (approval NMNSHP02-002).

## Additional Information

**How to cite this article**: Pike, D. A. *et al.* Surf and turf: predation by egg-eating snakes has led to the evolution of parental care in a terrestrial lizard. *Sci. Rep.* **6**, 22207; doi: 10.1038/srep22207 (2016).

## Supplementary Material

Supplementary Information

Supplementary Video

## References

[b1] Clutton-Brock T. H. The evolution of parental care. 13–29 (Princeton University Press, 1991).

[b2] Farmer C. G. Parental care: The key to understanding endothermy and other convergent features in birds and mammals. Am Nat. 155, 326–334 (2000). 10718729 10.1086/303323

[b3] Klug H. & Bonsall M. B. Life history and the evolution of parental care. Evolution 64, 823–835 (2009). 19796144 10.1111/j.1558-5646.2009.00854.x

[b4] Walling C. A., Stamper C. E., Smiseth P. T. & Moore A. J. The quantitative genetics of sex differences in parenting. Proc. Natl. Acad. Sci. USA 105, 18430–18435 (2008). 19008350 10.1073/pnas.0803146105 PMC2587554

[b5] Head M. L., Hinde C. A., Moore A. J. & Royle N. J. Correlated evolution in parental care in females but not males in response to selection on paternity assurance behaviour. Ecol. Lett. 17, 803–810 (2014). 24766255 10.1111/ele.12284 PMC4285953

[b6] Gross M. R. The evolution of parental care. Quart. Rev. Biol. 80, 37–46 (2005). 15884734 10.1086/431023

[b7] Shine R. In Biology of the Reptilia Vol. 16 (eds Gans C. & Huey R. B.) Ch. 4, 275–329 (Alan R. Liss, 1988).

[b8] Tallamy D. W. Insect parental care. Bioscience 1984, 20–24 (1984).

[b9] Wesolowski T. On the origin of parental care and the early evolution of male and female parental-roles in birds. Am Nat. 143, 39–58 (1994).

[b10] Gilbert J. D. & Manica A. The evolution of parental care in insects: A test of current hypotheses. Evolution 69, 1255–1270 (2015). 25825047 10.1111/evo.12656 PMC4529740

[b11] Gilbert J. D. & Manica A. Parental Care Trade‐Offs and Life‐History Relationships in Insects. Am. Nat. 176, 212–226 (2010). 20528469 10.1086/653661

[b12] Burley N. T. & Johnson K. The evolution of avian parental care. Phil. Trans. R. Soc. B 357, 241–250 (2002). 11958693 10.1098/rstb.2001.0923 PMC1692953

[b13] Gross M. R. & Sargent R. C. The evolution of male and female parental care in fishes. Am. Zool. 25, 807–822 (1985).

[b14] Klug H., Bonsall M. B. & Alonzo S. H. The orgin of parental care in relation to male and female life history. Ecol. Evol. 3 **(4)**, 779–791 (2013). 23610624 10.1002/ece3.493 PMC3631394

[b15] Reynolds J. D., Goodwin N. B. & Freckleton R. P. Evolutionary transitions in parental care and live bearing in vertebrates. Phil. Trans. R. Soc. B 357, 269–281 (2002). 11958696 10.1098/rstb.2001.0930 PMC1692951

[b16] Roff D. Life history evolution. 359–459 (Sinauer Associates, Inc, 2002).

[b17] Manica A. & Johnstone R. A. The evolution of paternal care with overlapping broods. Am. Nat. 164, 517–530 (2004). 15459882 10.1086/423792

[b18] Wilson E. O. Sociobiology: The new synthesis. 201–223 (Belknap Press of Harvard University, 1975).

[b19] Gordon C. E., Feit A., Grüber J. & Letnic M. Mesopredator suppression by an apex predator alleviates the risk of predation perceived by small prey. Proc. R. Soc. B 282, 20142870 (2015). 10.1098/rspb.2014.2870 PMC4344160 25652837

[b20] Stoks R., McPeek M. & Mitchell J. Evolution of prey behavior in response to changes in predation regime: damselflies in fish and dragonfly lakes. Evolution 57, 574–585 (2003). 12703947 10.1554/0014-3820(2003)057[0574:EOPBIR]2.0.CO;2

[b21] Stevens M. & Merilaita S. Animal camouflage: current issues and new perspectives. Phil. Trans. R. Soc. B 364, 423–427 (2009). 18990674 10.1098/rstb.2008.0217 PMC2674078

[b22] Mappes J., Marples N. & Endler J. A. The complex business of survival by aposematism. Trends Ecol. Evol. 20, 598–603 (2005). 16701442 10.1016/j.tree.2005.07.011

[b23] Tseng H.-Y., Lin C.-P., Hsu J.-Y., Pike D. A. & Huang W.-S. The functional significance of aposematic signals: geographic variation in the responses of widespread lizard predators to colourful invertebrate prey. PLoS ONE 9, e91777 (2014). 24614681 10.1371/journal.pone.0091777 PMC3948897

[b24] Speed M. P. When is mimicry good for predators? Anim. Behav. 46, 1246–1248 (1993).

[b25] Skelhorn J., Rowland H. M., Delf J., Speed M. P. & Ruxton G. D. Density-dependent predation influences the evolution and behavior of masquerading prey. Proc. Natl. Acad. Sci. USA 108, 6532–6536 (2011). 21464318 10.1073/pnas.1014629108 PMC3081003

[b26] Brown J. L., Morales V. & Summers K. A key ecological trait drove the evolution of biparental care and monogamy in an amphibian. Am Nat. 175, 436–446 (2010). 20180700 10.1086/650727

[b27] Webb J. N., Houston A. I., M. M. J. & Székely T. Multiple patterns of parental care. Anim. Behav. 58, 983–993 (1999). 10564600 10.1006/anbe.1999.1215

[b28] Huang W. S., Lin S. M., Dubey S. & Pike D. A. Predation Drives Interpopulation Differences in Parental Care Expression. J. Anim. Ecol. 82, 429–437 (2013). 23237108 10.1111/1365-2656.12015

[b29] Huang W. S., Greene H. W., Chang T. J. & Shine R. Territorial behavior in Taiwanese kukrisnakes (Oligodon formosanus). Proc. Natl. Acad. Sci. USA 108, 7455–7459 (2011). 21502515 10.1073/pnas.1101804108 PMC3088593

[b30] Huang W. S. & Pike D. A. Testing cost-benefit models of parental care evolution using lizard populations differing in the expression of care. PLoS ONE 8 e54065 (2013). 23408934 10.1371/journal.pone.0054065 PMC3567115

[b31] Coleman K., Rothfuss L. A., Ota H. & Kardong K. V. Kinematics of egg-eating by the specialized Taiwan snake Oligodon formosanus (Colubridae). J. Herpetol. 27, 320–327 (1993).

[b32] Huang W. S. Costs of egg caring in the skink, *Mabuya longicaudata*. Ecol. Res. 22, 659–664 (2007).

[b33] Helfield J. M. & Naiman R. J. Keystone interactions: salmon and bear in riparian forests of Alaska. Ecosystems 9, 167–180 (2006).

[b34] Lundberg J. & Moberg F. Mobile link organisms and ecosystem functioning: implications for ecosystem resilience and management. Ecosystems 6, 0087–0098 (2003).

[b35] Levi T. *et al.* Using grizzly bears to assess harvest-ecosystem tradeoffs in salmon fisheries. PLoS Biol. 10, e1001303 (2012). 22505845 10.1371/journal.pbio.1001303 PMC3323506

[b36] Hocking M. D. & Reynolds J. D. Impacts of salmon on riparian plant diversity. Science 331, 1609–1612 (2011). 21442794 10.1126/science.1201079

[b37] Huang W. S. & Pike D. A. Climate change impacts on fitness depend on nesting habitat in lizards. Funct. Ecol. 25, 1125–1136 (2011).

[b38] Huang W. S. Parental care in the long-tailed skink, *Mabuya longicaudata* on a tropical Asian island. Anim. behav. 72, 791–795 (2006).

[b39] Huang W. S. & Wang H. Y. Predation risks and anti-predation parental care behavior: an experimental study in a tropical skink. Ethology 115, 273–279 (2009).

[b40] Cheng I. J. e. a. Ten years of monitoring the nesting ecology of the green turtle, Chelonia mydas, on Lanyu (Orchid I.), Taiwan. Zool. Stud. 48, 83–94 (2009).

[b41] Gibbons J. W. & Andrews K. M. PIT tagging: simple technology at its best. Bioscience 54, 447–454 (2004).

[b42] Huang W. S. Resources exploitation by ants facilitates lizard egg survival. Ecol. Entomol. 33, 555–559 (2008).

[b43] Barton B. T. & Roth J. D. Implications of intraguild predation for sea turtle nest protection. Biol. Conserv. 141, 2139–2145 (2008).

[b44] Huang W. S. Foraging behaviors of two sympatric ant species in response to lizard eggs. Zoology 113, 85–90 (2010). 20199855 10.1016/j.zool.2009.06.003

