# Supplementary material for: Surf and turf: predation by egg-eating snakes has led to the evolution of parental care in a terrestrial lizard
Source: Sci Rep. 2016 Feb 26;6:22207. doi: 10.1038/srep22207 (PMC4768160; doi:10.1038/srep22207)
Supplement: Supplementary Information [file srep22207-s1.doc]

Surf and turf: predation by egg-eating snakes has led to the evolution of parental care in a terrestrial lizard

**Supplementary Information**

David A. Pike^1^, Rulon W. Clark^2^, Andrea Manica^3^, Hui-Yun Tseng^4^, Jung-Ya Hsu^4^ and Wen-San Huang^, 4,5*^

^1^ School of Marine and Tropical Biology, James Cook University, Australia

^2^Department of Biology, San Diego State University, USA

^3^Department of Zoology, University of Cambridge, CB2 3EJ, UK

^4^Department of Biology, National Museum of Natural Science, Taiwan

^5^Department of Life Sciences, National Chung Hsing University, Taiwan

^*^Correspondence: e-mail: wshuang@mail.nmns.edu.tw

**
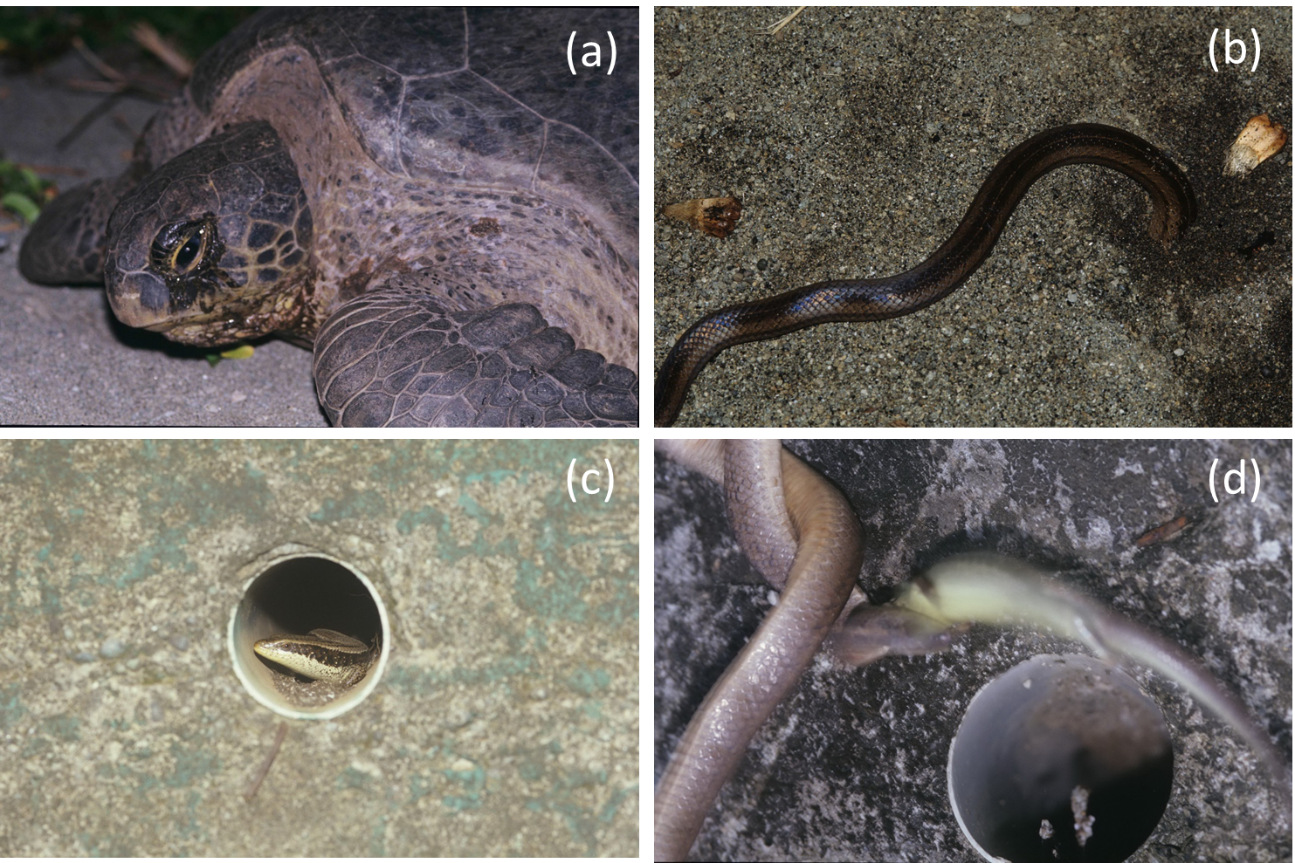
**

**Supplement Figure S1.** Predator-prey relationships on Orchid Island, Taiwan. Green sea turtles nest on sandy beaches (*a*), which egg-eating snakes locate and consume (*b*). Female long-tailed sun skinks guard their nests during egg incubation (*c*) to defend their nests from predation by egg-eating snakes (*d*). All photos by W.-S. H.


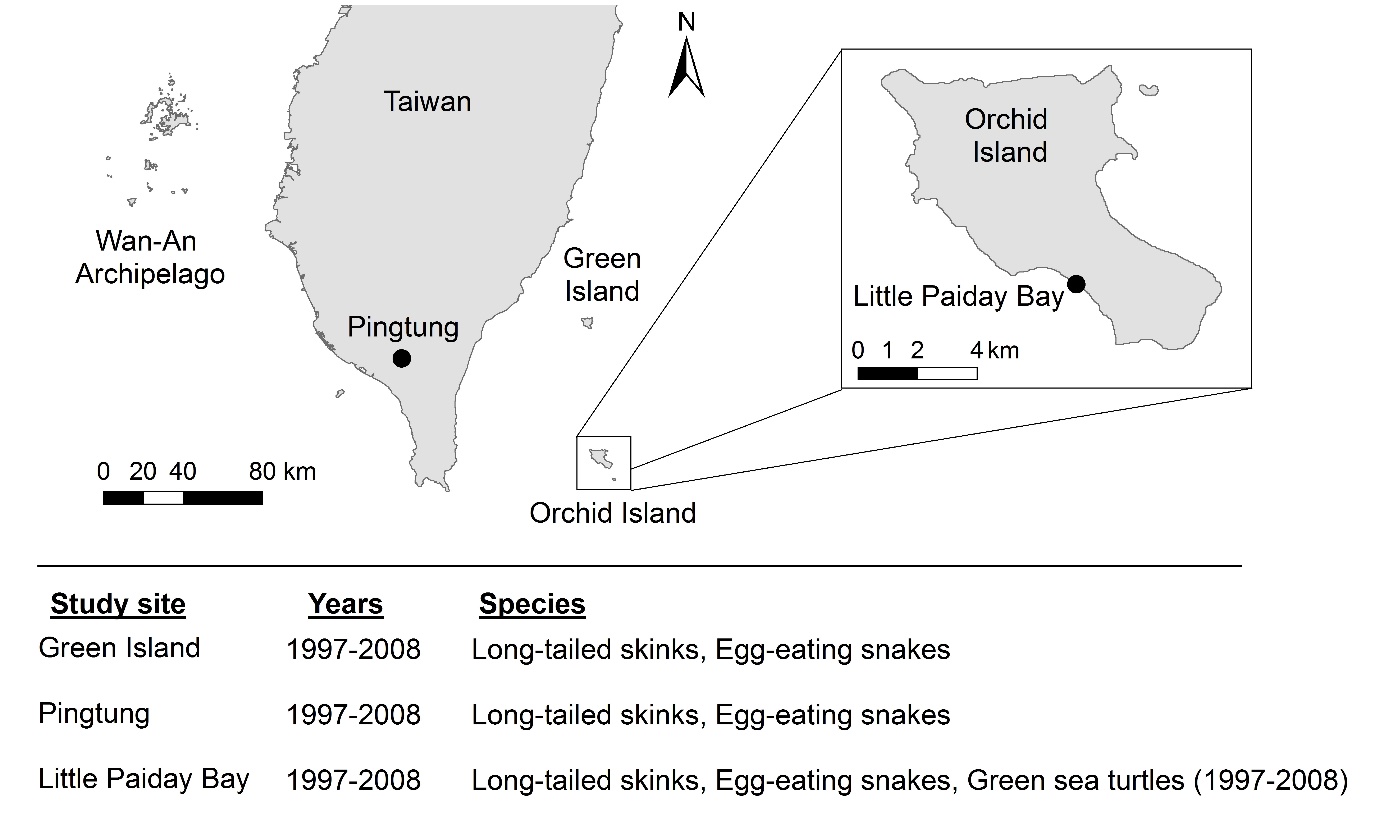


**Supplement Figure S2.** The species composition in three study localities during 1997-2008. The map was created using ArcMap Version 9.3 (http://en.softonic.com/s/arcmap-9.3-free-download).

| **Family** | **mainland Taiwan** | **Green Island** | **Orchid Island** |
| --- | --- | --- | --- |
| Agamidae | *Japalura swinhonis* | *Japalura swinhonis* | *Japalura swinhonis* |
| Gekkonidae | *Gekko hokouensis* | *Gekko hokouensis* | *Gekko hokouensis* |
|  |  |  | *G. kikuchii* |
|  | *Hemidactylus frenatus* | *Hemidactylus frenatus* | *Hemidactylus frenatus* |
|  |  | *Lepidodactylus lugubris* | *Lepidodactylus lugubris* |
|  |  |  | *Lepidodactylus yami* |
| Lacertidae | *Takydromus formosanus* | *Takydromus formosanus* |  |
|  |  | *Takydromus sauteri* | *Takydromus sauteri* |
| Scincidae |  | *Emoia atrocostata* | *Emoia atrocostata* |
|  |  |  | *Eutropis cumingi* |
|  | *Eutropis longicaudata* | *Eutropis longicaudata* | *Eutropis longicaudata* |
|  |  |  | *Eutropis multicarinata* |
|  |  | *Eumeces chinensis leucostictus* |  |
|  | *Sphenomorphus incognitus* | *Sphenomorphus incognitus* | *Sphenomorphus incognitus* |
| Colubridae | *Elaphe carinata*^1^ | *Elaphe carinata*^1^ | *Elaphe carinata*^1^ |
|  | *E. taeniura*^1^ |  |  |
|  | *Oligodon formosanus*^2^ | *Oligodon formosanus*^2^ | *Oligodon formosanus*^2^ |
|  | *Ptyas mucosus*^1^ |  |  |
| Elapidae | *Bungarus multicinctus*^1^ |  |  |
|  | *Naja naja atra*^1^ |  |  |
| Typhlopidae | *Typhlops braminus* | *Typhlops braminus* | *Typhlops braminus* |
| Viperidae | *Protobothrops mucrosquamatus*^1^ |  |  |
| Chelonidae |  |  | *Chelonia mydas*^3^ |
| Total species | 14 | 13 | 16 |

^1^lizard predator, ^2^egg predator, ^3^marine-dwelling

**Supplementary Table S1.** Oviparous reptiles occurring (and thus nesting) within 1km of our study sites. Lizard-eating endotherms are absent from the islands and *Oligodon formosanus* eats reptile eggs almost exclusively[^28^](#_ENREF_24).

**Video Legend**

The female long-tailed sun skink attacked the intruder *Oligodon formosanus* to protect its eggs.
